# Multiple mini-interviews as a predictor of academic achievements during the first 2 years of medical school

**DOI:** 10.1186/s13104-016-1866-0

**Published:** 2016-02-13

**Authors:** Hee Jae Lee, Sung Bae Park, Sung Chul Park, Won Sun Park, Sook-Won Ryu, Jeong Hee Yang, SungHun Na, Jun Yeon Won, Gi Bong Chae

**Affiliations:** School of Medicine, Kangwon National University, Hyoja-2, Chuncheon, Gangwon 200-701 Republic of Korea

**Keywords:** Multiple mini-interviews, Critical thinking, Professionalism, Communication skills

## Abstract

**Background:**

Recently, conventional interviews have been replaced with the multiple mini-interviews (MMI) for medical student selection in Korea. We first introduced the MMI as a new admissions tool in Korea. The aim of this study is to determine whether the MMI accurately predicts academic achievement on both written and performance-based examinations during the first 2 years of medical school.

**Methods:**

The original scores of each station were standardized to T-scores in the candidates group. Three cohorts of students were included depending upon the year they entered medical school. Pearson’s correlations were calculated to estimate the correlations between MMI scores and academic achievements. Additional correlated factors were run through multiple stepwise linear regression analysis to estimate predictive validity.

**Results:**

There were no differences between T-scores or grade point averages (GPA) among the cohorts. The correlation coefficients between total MMI scores and academic achievement in Year 1 and the Year 2 performance-based examinations ranged from 0.17 to 0.43. Station 1 significantly predicted academic achievement over the second year. Station 3 significantly predicted only performance-based examination performance over the second year.

**Conclusion:**

MMI is a useful tool to assist with medical student selection. In particular, critical thinking, professionalism, and presentation and communication skills may be meaningful topics for predicting academic achievements, especially in performance-based subjects.

## Background

In 2005 South Korea launched a medical graduate school system that is similar to those in the United States (US) and Canada. Following this conversion admissions tools also needed to be modified. Previously, the college scholastic ability test (CSAT), similar to the scholastic aptitude test (SAT), comprised the majority of the assessment factors for admission to medical schools. However, since medical school candidates were now college graduates rather than only high school graduates, new assessment methods were required for selection. Today, most of the medical schools in South Korea use medical education eligibility test (MEET) scores, undergraduate grade-point averages (uGPAs), English proficiency test scores, and conventional admission interviews. Although cognitive admission variables have shown high face validity and reliability in pre-clinical academic performance [1–3], assessing a candidate’s non-cognitive characteristics is crucial in the admission process to medical school as well. Therefore, we were the first medical school to adopt multiple mini-interviews (MMI) as part of the admissions interview process to select medical students in South Korea [4].

The MMI is accepted worldwide as a specific form of admissions interview to assess the non-cognitive characteristics of candidates in health science fields [5–7]. The merit of the MMI is its reliability and cost efficiency compared with traditional interview formats [6]. However, few studies have shown the predictive validity of MMIs regarding academic achievement during medical school [8–10]. Reiter et al. [8] demonstrated that MMIs correlate significantly with the clerkship director’s rating (r = 0.57) and encounter cards (r = 0.51). Recently, MMIs were shown to have significant positive correlations with both written exams and objective structured clinical examinations across two separate cohorts and years [9]. However, the data were insufficient to support the predictive validity of MMIs related to in-program performance.

We previously reported the reliability of MMIs based on their generalizability [4]. Our MMI circuit had three stations, each lasting 8 min, with a 2 min gap between stations. In addition, we illustrated that MMI scores can predict medical students’ academic achievements in the medical humanities and in clinical practice [11]. However, this study has the limitations that the number of enrolled students was small, only 46 members, and participants were from a single cohort. Therefore, we unified the topics and process of the MMI format and reviewed data from the 2011–2013 students admitted to medical school. The aim of this study was to determine whether MMIs could predict academic achievements during the first 2 years of medical school at Kangwon National University (KNU).

## Methods

### Participants

The participants were students who entered KNU Medical School during the years 2011–2013 (Table 1). They were selected among potential candidates by using several assessment tools. In brief, 29, 31 and 32 students participated from each year during 2011–2013. Thus, totally 92 students participated in the first year of medical school. Since their GPAs fell below 2.0 on a 4.5 point scale, eight participants were excluded in the second year (N = 84). There were no differences in the mean ages among the entrance years.

**Table 1:** Demographic characteristics of the candidates and participants who entered medical school in 2011–2013

Characteristic	2011	2012	2013	Total
Candidates (applied)				
No.	80	80	89	249
Age, years (SD)	26.6 (2.98)	25.5 (2.84)	24.7 (3.23)	25.6 (3.12)
Gender, M/F %	52.5/47.5	57.5/42.5	59.6/42.5	56.6/43.4
Participants (admitted)				
No.	29	31	32	92
Age, years (SD)	26.1 (2.95)	26.0 (3.15)	25.8 (4.13)	26.0 (3.44)
Gender, M/F %	48.3/51.7	54.8/45.2	56.3/43.8	53.3/46.7
Year 1	29	31	32	92
Year 2	26	28	30	84

*SD* standard deviation, *M* male, *F* female

### Admission procedures

KNU Medical School’s admission process is coordinated in two steps. In the first step, the MEET score, uGPA and the score on an English proficiency test such as the Test of English as a Foreign Language (TOEFL) are evaluated to assess the candidate’s cognitive characteristics. In the next step, 80 and 89 candidates (Table 1) were selected to participate in MMIs in 2011–2013.

For the MMIs, candidates rotated through three stations (8 min per station) and were evaluated by one interviewer at each station. All interviewers had to attend an MMI workshop, which included MMI concepts and interview skills. Furthermore, interviewers had multiple practice runs with simulated students to practice standardizing scoring on the day before each MMI. The topics at Station 1 were critical thinking and presentation skills. A month earlier, we had announced selected readings such as Critical Reasoning in Ethics by Anne Thomson, Why Morality by Michael J. Sandel, and Justice by Michael J. Sandel to the candidates in 2011, 2012 and 2013 to help them prepare for the interviews. For Station 1, the candidates were given 30 min to prepare a presentation about ethical issues related to those books. Next, ethical problem solving and professionalism including communication skills were assessed at Station 2 and Station 3, respectively. Each candidate’s score was standardized to a T-score at each station. Finally, successful applicants were selected based on their overall score in step one and their MMI scores.

### Medical school examination

During the first year of the medical school curriculum, applicants must take 43 credits of basic medical sciences courses, as well as research overview, medical interview and history taking courses, which are each one credit. In the second year, students need to take 34 credits of integrated medicine courses, three credits of medical humanities and social science courses, and five credits of a basic clinical procedures course. Two assessment tools, written and performance-based tests, were used to evaluate the students’ academic achievements in each course through the relative grading system.

### Statistical analysis

All data were analyzed with SPSS Statistics 21 for Windows (IBM, Armonk, NY, USA). One-way analysis of variance (ANOVA), following Bonferroni post hoc tests, was performed to compare the differences in ages, total MMI scores, T-scores at each station, and GPAs in medical courses for 2 years among the cohorts. To confirm the correlation between the admission tools and academic achievements in medical courses over 2 years, Pearson’s correlations were calculated. Then, multiple stepwise linear regression analyses were performed to determine whether the admissions tools predicted academic achievements in medical courses over 2 years. Values were considered statistically significant when P was <0.05. Because previous studies shown that more male students have relatively lower grades on academic achievement compared with female students [12, 13], the significance levels were adjusted for gender at the time of testing.

### Ethical approval

This study was approved by IRB in Kangwon National University for both review and informed consent exemption according to South Korea’s law of Bioethics and Safety Act (KWNUIRB-2015-11-003).

## Results

According to the one-way ANOVA, there was no difference in the participants’ GPAs comparing semester data or between examination types among entrance years (Table 2). Additionally, each participant’s T-scores for each station by entrance years were similar (Table 3). Thus, we decided to sum the 3 years’ data for further analysis.

**Table 2:** Comparison of academic achievements in medical courses by entrance year for 2 years

GPA (SD)	2011	2012	2013	Total
Year 1				
Semester 1	3.05 (0.83)	3.08 (0.78)	3.08 (0.84)	3.07 (0.81)
Semester 2	3.17 (0.63)	3.23 (0.69)	3.30 (0.63)	3.23 (0.65)
Written	3.10 (0.70)	3.17 (0.68)	3.21 (0.70)	3.16 (0.69)
Performance-based	3.32 (0.57)	3.55 (0.56)	3.51 (0.53)	3.46 (0.56)
Year 2				
Semester 1	3.06 (0.58)	2.98 (0.59)	2.88 (0.58)	2.97 (0.58)
Semester 2	2.92 (0.74)	3.00 (0.61)	2.99 (0.73)	2.97 (0.69)
Written	3.22 (0.71)	3.18 (0.66)	3.12 (0.76)	3.17 (0.70)
Performance-based	2.92 (0.52)	3.00 (0.48)	2.98 (0.48)	2.98 (0.48)

*GPA* grade point average, *SD* standard deviation

**Table 3:** Station scores for participants by entrance year

	2011	2012	2013	Total
MMI total scores, mean (SD)	152.2 (16.21)	151.3 (12.79)	155.0 (16.40)	152.9 (15.12)
Station 1, mean T-scores (SD)				
Critical thinking, presentation	52.6 (9.93)	50.0 (8.94)	51.2 (8.94)	51.2 (9.33)
Station 2, mean T-scores (SD)				
Ethical problem solving	50.2 (8.86)	51.4 (8.64)	51.1 (8.64)	50.9 (8.38)
Station 3, mean T-scores (SD)				
Professionalism, communication	49.4 (8.77)	49.9 (8.75)	52.6 (10.14)	50.7 (9.28)

*MMI* multiple mini-interview, *SD* standard deviation

### Admissions tools and academic achievements: Year 1

In the first year, 92 students were enrolled. The participants had completed 45 credits of courses, 43 graded by written examinations and two graded by performance-based exams. Statistically significant correlations ranged from 0.174 to 0.392 (Table 4). Total MMI scores and uGPAs showed significantly positive correlations with overall GPA and results of both written examinations and performance-based tests. However, there were no significant correlations between the results of the performance-based examinations and the MEET scores. The US Department of Labor has published guidelines about interpreting the strengths of correlations between the test and a measure of job criterion (validity coefficients <0.11, “unlikely to be useful”; 0.11–0.20, “dependent on circumstance”; 0.21–0.35, “likely to be useful”; >0.35, “very beneficial”) [14]. Based on these guidelines, the validity coefficient for the MEET scores was “very beneficial”, and those for the MMI score and uGPA were “likely to be useful”.

**Table 4:** Correlations between admissions tools and academic achievements in medical courses for 2 years

Academic achievements (GPA)		Credit	MEET	UGPA	MMI			
					Station 1	Station 2	Station 3	Total
Year 1	Total	45	.387**	.269**	.096	.142	.158	.235*
(N = 92)	Written	43	.393**	.272**	.091	.143	.151	.228*
	Performance	2	−.002	.175*	.116	−.003	.171	.174*
Year 2	Total	42	.254*	.187*	.216*	−.131	.081	.118
(N = 84)	Written	32	.265**	.166	.191*	−.130	.012	.060
	Performance	10	.023	.163	.291**	−.069	.452**	.427**

The significant levels were adjusted for gender at the time of analysis

*uGPA* undergraduate grade point average, *MEET* medical education eligibility test, *MMI* multiple mini-interview

* P < 0.05, ** P < 0.01 significant correlation

Multiple stepwise linear regression analyses were performed to determine the accuracy of the MEET scores, uGPAs and total MMI scores in predicting GPA in the first year (Table 5). The linear combination of MEET scores, uGPAs and total MMI scores was significantly related to total GPA [F (3, 88) = 10.252, P < 0.001] and the GPA from written examinations [F (3, 88) = 10.397, P < 0.001]. These values explained approximately 26 % of the variance between the first-year GPA and the written examinations. The regression equation for predicting the total GPA was: total GPA = 0.336 × MEET score + 0.248 x uGPA score + 0.217 × MMI score − 4.931. In addition the regression equation for predicting the written exams GPA was: written exams GPA = 0.342 × MEET score + 0.252 × uGPA score + 0.208 × MMI score − 5.399.

**Table 5:** Multiple stepwise linear regression statistics

Academic achievements (GPA)		*R* ^*2*^	*F*	*P*	Predictor	*B*	*SE*	*ß*	*P*
Year 1	Total	0.259	10.252	<0.001	MEET	0.028	0.008	0.336	0.001
					uGPA	0.036	0.013	0.248	0.009
					MMI total	0.009	0.004	0.217	0.020
	Written	0.262	10.397	<0.001	MEET	0.030	0.008	0.342	<0.001
					uGPA	0.038	0.014	0.252	0.008
					MMI total	0.009	0.004	0.208	0.026
Year 2	Total	0.065	5.642	0.020	MEET	0.019	0.008	0.254	0.020
		.0470	4.032	0.048	Station 1	0.012	0.006	0.216	0.048
	Written	0.070	6.182	0.015	MEET	0.023	0.009	0.265	0.015
	Performance	0.182	18.252	<0.001	MMI total	0.013	0.003	0.427	<0.001
		0.278	15.573	<0.001	Station 1	0.013	0.005	0.271	0.005
					Station 3	0.023	0.005	0.440	<0.001

The significant levels were adjusted for gender at the time of analysis

*uGPA* undergraduate grade point average, MEET medical education eligibility test, *MMI* multiple mini-interview

### Admissions tools and academic achievements: Year 2

If a students’ GPA is not over 2.0, they must repeat the first year program for another year. Of this original cohort eight students needed to remain in first year courses and 84 students were enrolled in the second year. The second year participants completed 42 credits of courses, of which 32 credits were graded by written examination and ten by performance-based exam. Statistically significant correlations ranged from 0.187 to 0.452 (Table 4). As was shown in the first year, the MEET score and uGPA score significantly correlated with second-year GPA. There was no correlation between total MMI score. Interestingly, Station 1, which assessed critical thinking ability and presentation skills, significantly correlated with the final second-year GPA. Furthermore, even though there was no correlation between the second year-GPA and MMI total score, total MMI was significantly correlated to the results from the performance-based examinations. The validity coefficient for the MMI total scores was 0.427, which means that the MMI is very beneficial admission tool. Typically, Station 1 (Critical Thinking and Presentation Skills) and Station 3 (Professionalism and Communication Skills) showed significant correlations to the performance-based exam results. The magnitude of the correlation for Station 3 (0.452) was higher than that for any of the other admissions tools.

We performed multiple stepwise linear regression analyses to determine the contributions of MEET score, uGPA and total MMI score in predicting final second-year GPA (Table 5). The MEET score (β = 0.254, P = 0.020) and Station 1 score (β = 0.216, P = 0.048) were positively correlated with the second-year GPA. Interestingly, only the MMI total score (β = 0.427, P < 0.001) significantly predicted the GPAs for the performance-based exams. It explained approximately 18 % of the variance in the GPAs for the performance-based examinations. The scores for each MMI station explained approximately 28 % of the variance between the GPAs for the performance-based examinations. The regression equation for predicting the GPA for the performance-based examinations was: GPA = 0.44 × Station 3 score + 0.271 × station 1 score + 1.883.

## Discussion

Today, the MMI is known as a reliable and reputable admissions tool to assess non-cognitive attributes. More than 40 previous studies suggested the reliability of the MMI, but only a few studies reported its predictive validity regarding academic success [6]. This study explored the relationship between admissions tools and academic achievement for the first 2 years of medical school.

In other nations, entrance exam scores predict first-year medical school GPA [9, 15–17]. As with these previous results [1], we also showed statistically significant correlations between MEET score both at first and second-year GPA. However, both cognitive and non-cognitive attributes, such as critical thinking skills and the motivation for medical professionalism, were found to be significant predictors of academic success during the first year of medical school [18–20]. Furthermore, a study by Husbands and Dowell [9] found that MMI scores predict written exam scores in the first 2 years of medical school. Our study also demonstrated that the MMI showed significant positive correlations with written exam scores in the first year. However, these results present limited evidence for the predictive validity of the MMI because the first-year curriculum at the KNU Medical School is about basic medical science and each course includes experimental practice. Even though only 5–10 % of the grades for each course reflected performance-based assessment, total MMI scores may effectively predict performance on written assessments in the first year; there were no correlations between total MMI scores and MEET (r = 0.07; P = 0.562) scores or uGPA (r = 0.08; P = 0.461). Otherwise, negative correlation or non-significant results of correlation between non-cognitive attributes and academic achievement at a medical school were reported [21, 22]. However, we showed that the MMI total score significantly correlated to academic achievements based on performance-based tests both in the first and second years. The courses called Research Overview, Integrated Medicine, Doctoring and Medical Humanities I, Problem-based Learning, Technical and Procedure Skills, and Physical Examination were evaluated by performance-based tests. Furthermore, we reported that the MMI score correlated to academic achievement in subjects in the medical humanities and clinical practice [23]. Thus, our results also supported that non-cognitive attributes could predict academic success during the earlier year of medical school.

A number of studies have shown that the McMaster University MMI scores have consistently predicted in-program and licensing examination performance [6, 8, 24, 25]. These studies have also demonstrated that the MMI is one of the significant predictors for evaluating post-graduation performance. As with Husbands and Dowell [9], we found that total MMI scores correlated with performance-based exams during the first and second years. In particular, critical thinking, presentation, professionalism, and communication skills might be important competences in performance-based academic achievement. It has been shown that critical thinking skills predict academic success during the preclinical years of medical education [18]. Critical thinking involves logical analysis, active reasoning, problem solving, and appropriate decision making. At Station 1, critical thinking and presentation skills were primarily evaluated. A month earlier, the candidates were encouraged to read one of our suggested books, such as Critical Reasoning in Ethics by Anne Thomson or Why Morality or Justice by Michael J. Sandel, and prepare for their interview. During the interview, the candidates needed to prepare a presentation about their opinion about ethical issues discussed in those books. Interestingly, we showed that the Station 1 score could predict the results of written and performance-based exam in second year. However, there was no significance in the results of written test in first year. This might be due to the fact that first year tests are highly focused on basic medicine skills and not medical reasoning. While not only knowledge but also reasoning oriented of clinical medicine courses were assessed in second year, both written and performance based results could be predicted by Station 1 results. In the case of Station 3, we primarily assessed professionalism including communication skills, self-understanding and motivation. We found that Station 3 score most highly correlated with performance-based exams (r = 0.452, *P* < 0.001). Especially, correlations were largest in Doctoring Medical Humanities (DMH) I course (r = 0.42, *P* < 0.001). This course is about developing medical professionalism. Personal development, such as self-awareness, self-confidence, self-regulation, motivation and career choice, is one of the main virtues in medical professionalism. According to multiple regression analysis, Station 3 scores explained approximately 24 % of the variance in DMH I course (*F* = 6.087, model *P* < 0.001; *β* = 4.774, Stand *β* = 0.348, *P* = 0.002). Therefore, considering non-cognitive attributes, especially communication skills, self-understanding and motivation, have some validity to predict academic achievement on course for developing medical professionalism.

This study had a number of limitations. The first was the small number of participants admitted each year. According to statistical power analyses using G* Power 3.1 [26], the minimum required sample size was 77 for multiple linear regression in this study [anticipated effect size (f^2^) = 0.15; desired statistical power level = 0.8; probability level = 0.05]. Although the number of participants in a single year was less than the minimum sample size, a sum of the number of participants from all 3 years was over than 77, (starting with 92 and ending with 84 participants). Second, the degree of difficulty and the reliability of each station could have contributed biases. Also, three MMI stations could be unsuitable to reduced unwanted variances [27]. Increasing the number of stations, to more than six stations, might impact future reliability [6]. To reduce systematic or unsystematic rater variance, however, improving the quality of the interviewer training program would be beneficial [6, 28]. Before participating in the MMI, interviewers had to complete four steps for this study. First, they have to attend the interviewer workshop to learn about the MMI and participate in a mock trail. Second, the day before MMI, they received the original script and evaluation card. They gathered together to discuss and adjust the criterion of assessment. Next, they performed preliminary exercises by themselves. Finally, they had a rehearsal to reconfirm the criterion of assessment with four mock candidates. This kind of interviewer training might increase the reliability of our study. Third, we did not consider the influence of the students’ activities and lifestyles while they were in medical school. Several studies showed that social factors and emotional stability correlated to academic achievement in medical students [12, 13]. Despite these limitations, this study is meaningful because it replicated Husbands and Dowell [9] results that MMI correlated to academic achievement in early years at medical school.

## Conclusions

Medial schools want to identify and admit optimal candidates who will perform effectively during in medical school and be a good doctor in the future. Even though this small sample size and three MMI stations are the limitations of this study, we confirmed that MEET and uGPA are good pre-admission predictors for academic success in the early years of medical school. Also, the MMI could be reliable and acceptable as a predictor of achievement for the first 2 years of medical school. In particular, critical thinking, presentation, professionalism, and communication skill may be meaningful topics for predicting academic achievements, especially in performance-based subjects. Additional research has been prearranged to predict the correlation between the MMI and academic achievement in subjects of clinical practice.
